# Study protocol for a systematic review of evidence for lifestyle interventions targeting smoking, sleep, alcohol/other drug use, physical activity, and healthy diet in people with bipolar disorder

**DOI:** 10.1186/s13643-016-0282-9

**Published:** 2016-07-05

**Authors:** Frances J. Kay-Lambkin, Louise Thornton, Julia M. Lappin, Tanya Hanstock, Louisa Sylvia, Felice Jacka, Amanda L. Baker, Michal Berk, Phillip B. Mitchell, Robin Callister, Naomi Rogers, Stephanie Webster, Simon Dennis, Christopher Oldmeadow, Andrew MacKinnon, Christopher Doran, Alyna Turner, Sally Hunt

**Affiliations:** National Health and Medical Research Council Centre for Research Excellence in Mental Health and Substance Use, National Drug and Alcohol Research Centre, University of New South Wales, Sydney, Australia; Priority Research Centre for Translational Neuroscience and Mental Health, The University of Newcastle, Newcastle, Australia; Black Dog Institute, School of Psychiatry, University of New South Wales, Sydney, Australia; Department of Psychiatry, Harvard Medical School, Boston, USA; IMPACT Strategic Research Centre (Innovation in Mental and Physical Health and Clinical Treatment), Deakin University, Waurn Ponds, Australia; School of Biomedical Sciences and Pharmacy, Faculty of Medicine, The University of Newcastle, Newcastle, Australia; Brain and Mind Institute, University of Sydney, Sydney, Australia; CReDITSS, Hunter Medical Research Institute, Newcastle, Australia; The University of Melbourne, Melbourne, Australia; School of Psychology, University of Newcastle, Newcastle, Australia

**Keywords:** Bipolar disorder, Risk, Prevention, Mania, Depression, Tobacco, Smoking, Physical activity, Sleep, Diet, Weight loss, Alcohol/other drug use, Interventions, Treatment, Morbidity, Mortality

## Abstract

**Background:**

People with bipolar disorder (BD) have a mortality gap of up to 20 years compared to the general population. Physical conditions, such as cardiovascular disease (CVD) and cancer, cause the majority of excess deaths in psychiatric populations and are the leading causes of mortality in people with BD. However, comparatively little attention has been paid to reducing the risk of physical conditions in psychiatric populations. Unhealthy lifestyle behaviors are among the potentially modifiable risk factors for a range of commonly comorbid chronic medical conditions, including CVD, diabetes, and obesity. This systematic review will identify and evaluate the available evidence for effective interventions to reduce risk and promote healthy lifestyle behaviors in BD.

**Methods/design:**

We will search MEDLINE, Embase, PsychINFO, Cochrane Database of Systematic Reviews, and CINAHL for published research studies (with at least an abstract published in English) that evaluate behavioral or psychosocial interventions to address the following lifestyle factors in people with BD: tobacco use, physical inactivity, unhealthy diet, overweight or obesity, sleep-wake disturbance, and alcohol/other drug use. Primary outcomes for the review will be changes in tobacco use, level of physical activity, diet quality, sleep quality, alcohol use, and illicit drug use. Data on each primary outcome will be synthesized across available studies in that lifestyle area (e.g., tobacco abstinence, cigarettes smoked per day), and panel of research and clinical experts in each of the target lifestyle behaviors and those experienced with clinical and research with individuals with BD will determine how best to represent data related to that primary outcome. Seven members of the systematic review team will extract data, synthesize the evidence, and rate it for quality. Evidence will be synthesized via a narrative description of the behavioral interventions and their effectiveness in improving the healthy lifestyle behaviors in people with BD.

**Discussion:**

The planned review will synthesize and evaluate the available evidence regarding the behavioral or psychosocial treatment of lifestyle-related behaviors in people with BD. From this review, we will identify gaps in our existing knowledge and research evidence about the management of unhealthy lifestyle behaviors in people with BD. We will also identify potential opportunities to address lifestyle behaviors in BD, with a view to reducing the burden of physical ill-health in this population.

**Systematic review registration:**

PROSPERO CRD42015019993

**Electronic supplementary material:**

The online version of this article (doi:10.1186/s13643-016-0282-9) contains supplementary material, which is available to authorized users.

## Background

People with mental illness have a reduced life expectancy of between 12 and 20 years compared to the general population [1, 2]. To date, much of the attention on this issue has focused on the increased risks of suicide in people with mental illness, which has been shown to account for about 20 % of the premature deaths in populations with a mental illness. Comparatively little attention has been paid to reducing the risk of physical conditions, which account for 80 % of excess deaths in psychiatric populations [2].

A particular focus on bipolar disorder (BD) is important. In BD, reduced life expectancy in comparison with the general population ranges from 11 to 20 years depending on the age of onset of the disorder [3]. The reasons for this disparity are complex and multi-faceted, involving genetic, biological, environmental, psychological, and social factors. Cardiovascular disease (CVD) diagnoses occur 6 years earlier for those with BD than for depression and 15 years ahead of the general population [4]. Many factors are attributed to this, including the significant and adverse effects of medication for BD, such as weight gain, metabolic syndrome, high cholesterol, hypertriglyceridemia, and other cardiometabolic problems [5]. Medications commonly prescribed in BD—anti-manic, antipsychotic, and antidepressant treatments—can negatively contribute through adverse side effects including weight gain, sedation, and disordered glucose tolerance [6]. In addition, the phases of BD mean that people can oscillate between intense periods of severe depression (with high risk of suicide) followed by intense periods of activity and irritability, masking a cycle of agitation, sleep, and physical exhaustion that can have a significant impact on confidence, self-efficacy, and health-related decisions [7]. Up to 65 % of people with BD report current tobacco use [8] and smoke at 2.6 times the rate of people with major depressive disorder (MDD) and 6.3 times more than the general population [9]. Smoking is independently associated with suicide risk, poorer response to treatment, and structural brain changes [10]. Physical inactivity has been identified as an independent predictor of premature mortality among people with BD and significantly increases their risk of CVD [11]. In a study of 1046 Australian women, poor diet quality was associated with twice the odds for BD [12]. In another study comparing 2032 participants with BD in the general population, having BD was associated with significantly poorer eating behaviors, including fewer daily meals and difficulty obtaining or cooking food, as well as increased appetite and caloric intake [13]. Complicating this and supporting an integrated approach, people at risk for one adverse health behavior are at greater risk for others [14]. There is often poor integration between mental health and physical health services internationally and well-documented concerns by people with mental illness using existing services, who report inequity of access to physical-health-related interventions and support [9]. Together, the periods of being unwell with a psychiatric illness, combined with side effects of many medications, may contribute to the inequality in physical wellness for clients with mental health issues and the general population.

In order to reduce the mortality gap between people with BD and the general population, greater understanding is required of how best to assist them and their clinicians to address the potentially modifiable lifestyle behaviors associated with this gap [15, 16]. Recently, the International Society for Bipolar Disorder (ISBD) developed consensus guidelines for monitoring of adverse side effects associated with commonly prescribed medications for the disorder [17]. The aim for these guidelines was to increase the acknowledgement and focus of health care providers on the physical health comorbidities germane to the management of BD [17]. Whilst this is critically important to the field, reducing the medication load for people with BD is just one strategy in addressing the mortality gap associated with the condition [18].

Of additional importance is to identify acceptable and effective interventions to improve the health outcomes of people with BD. There is increasing recognition of the importance of supporting all people with mental health problems to focus on lifestyle factors, such as smoking cessation, in addition to symptom management [19]. However, the nature of BD may mean that additional strategies are indicated to ensure safety and well as efficacy of these approaches. In contrast to other mental disorders, for example, regulation and structure are critical in BD for both symptom and lifestyle management. For example, when depressed, strategies need to encourage stimulation and activation; however, when manic, the opposite is true [20]. Key withdrawal symptoms of nicotine are insomnia and increased appetite, with standard behavioral programs recommending increased physical activity to cope with cravings, stress, and weight gain [21]. Sleep disturbance, increased appetite, and excessive exercise are all critical warning signs for a relapse to BD [22], and thus careful strategies for self-regulation may be a unique feature of any program that recommends changing these behaviors in people with BD. To date, no systematic review has examined these issues or the available evidence to support lifestyle management in people with BD specifically.

The objective of this review is to systematically identify and evaluate the literature regarding lifestyle behavioral interventions among people with BD. We aim to synthesize the available evidence regarding the management of unhealthy lifestyle behaviors and the promotion of health behaviors in people with BD, with regard to the impact of such interventions on health, symptoms, and related outcomes. Specifically, we aim to answer the following questions:

- Which, if any, interventions have demonstrated effectiveness in the treatment of tobacco use, sleep-wake disturbances, alcohol/other drug use, or in encouraging healthy eating or healthy levels of physical activity for people with BD?
- Do any integrated evidence-based interventions exist that target a range of lifestyle behaviors within one package for people with BD? If so, do these integrated treatments produce improvements across all of the treatment targets?
- Have any lifestyle-based interventions been tested in people with BD that incorporate eHealth (and related technologies)? If so, how have outcomes compared with face-to-face delivered interventions?

## Methods

This systematic review is registered with the International Prospective Register of Systematic Reviews (PROSPERO, http://www.crd.york.ac.uk/PROSPERO, registration number: CRD42015019993). The protocol has been written according to the Preferred Reporting Items for Systematic Reviews and Meta-Analyses-Protocol (PRISMA-P) recommendations for systematic review protocols [23], and the findings will be reported using PRISMA guidelines [24].

### Criteria for study inclusion

To be eligible for inclusion in the planned review, articles will be required to describe an intervention conducted in people with BD aged 18 years and over. Any type of behavioral or psychosocial intervention will be considered; however, articles must report at least one behavioral health or lifestyle outcome (e.g., smoking, alcohol or other substance use, diet, physical activity, sleep). Additionally, eligible articles will be required to report their results for participants with bipolar disorder separately to any other participants in the study, be published in a peer-reviewed journal, and published in English (at least an abstract). Randomized trials as well as uncontrolled trials including cohort, historical, and case-control studies will be considered. Our primary outcomes of interest will be the following lifestyle behavior outcomes: tobacco use, physical activity, diet quality, sleep quality, alcohol use, cannabis use, and illicit drug use from baseline to the last available follow-up. Secondary outcomes of interest will include participants’ psychiatric symptoms and physical health outcomes such as changes in quality of life scores, depression, manic symptoms, body mass index, and other CVD risk factors from baseline to the last available follow-up as available.

### Search strategy for identification of studies

We will search the following electronic databases to identify potentially eligible studies for inclusion in the review: MEDLINE, Embase, PsychINFO, Cochrane Database of Systematic Reviews, and CINAHL. The search will be limited to human research and those with at least an abstract written in English. No date restrictions will be placed on the searches, and search terms will be intentionally kept broad and general to increase the likelihood of capturing potentially eligible studies. An example of the proposed search strategy for MEDLINE is outlined in Table 1.

**Table 1:** Search strategy using MEDLINE 1946 to present with daily update

Number	Searches	Results
1	Bipolar Disorder/	33,501
2	bipolar*.tw.	43,114
3	affective disorder*.mp. or Mood Disorders/	24,504
4	manic*.tw.	9456
5	mania.tw.	7439
6	hypomani*.tw.	2075
7	(mixed adj (state* or episode*)).mp. [mp = title, abstract, original title, name of substance word, subject heading word, keyword heading word, protocol supplementary concept word, rare disease supplementary concept word, unique identifier]	1096
8	Cyclothymic Disorder/	565
9	cyclothymi*.tw.	751
10	rapid next cycl*.tw.	0
11	schizoaffective.tw.	4195
12	Affective Disorders, Psychotic/or Psychotic Disorders/	40,311
13	unipolar*.tw.	8387
14	1 or 2 or 3 or 4 or 5 or 6 or 7 or 8 or 9 or 10 or 11 or 12 or 13	117,677
15	Smoking/or Marijuana Smoking/or Smoking Cessation/	137,906
16	"Tobacco Use"/	394
17	(smok* or tobacco or cigarette* or nicotine).tw.	244,392
18	exp Alcohol Drinking/	55,319
19	alcoholic intoxication/or alcoholism/	78,084
20	(alcohol* or drinking).tw.	281,327
21	marijuana abuse/or exp opioid-related disorders/	24,563
22	Cannabis/	7067
23	designer drugs/or exp street drugs/	10,732
24	exp Sleep/	64,880
25	sleep.tw.	104,983
26	Exercise/or Exercise Therapy/	101,251
27	Physical Exertion/	53,748
28	Motor Activity/	83,896
29	Sports/	25,382
30	(Physical Education and Training).mp. [mp = title, abstract, original title, name of substance word, subject heading word, keyword heading word, protocol supplementary concept word, rare disease supplementary concept word, unique identifier]	12,568
31	exp Diet Therapy/	44,656
32	(diet* adj5 (health* or weight*)).tw.	26,065
33	(calorie adj5 (control or reduc* or restrict*)).tw.	3102
34	food choice*.tw.	2516
35	(exercise* or sport*).tw.	232,095
36	(physical activit* or physical inactivit*).tw.	62,477
37	nutrition*.tw.	179,639
38	Sedentary Lifestyle/	4056
39	(sedentary adj5 (lifestyle or behavio?r*)).tw.	4236
40	(weight adj (loss or reduc* or gain* or change*)).tw.	103,986
41	(drug adj (use* or abus*)).tw.	56,661
42	(diet* adj5 quality).tw.	4443
43	15 or 16 or 17 or 18 or 19 or 20 or 21 or 22 or 23 or 24 or 25 or 26 or 27 or 28 or 29 or 30 or 31 or 32 or 33 or 34 or 35 or 36 or 37 or 38 or 39 or 40 or 41 or 42	1,374,954
44	random*.tw.	711,373
45	cross-over studies/or double-blind method/or random allocation/or single-blind method/	248,804
46	Randomized Controlled Trial/	404,345
47	Controlled Clinical Trial/	89,970
48	trial.tw.	368,603
49	groups.tw.	1,376,585
50	(evaluation* or treatment* or intervention*).tw.	4,060,728
51	44 or 45 or 46 or 47 or 48 or 49 or 50	5,446,183
52	14 and 43 and 51	6410
53	limit 52 to english language	5828

NOTE: use of the * in this table is to ensure that all derivations of the search term are captured in the search strategy

### Data extraction

Following de-duplication of the search results, two team members (LT and FK-L) will independently review the titles and abstracts of all returned articles to identify studies that potentially meet the inclusion criteria. Potentially eligible articles will then be reviewed in full by two team members against the eligibility criteria (LT and FK-L). Any disagreement between the team members regarding eligibility will be resolved through discussion with a third reviewer (LS). Reasons why studies were excluded from this phase of the search will be recorded.

Trial reports (e.g., from clinical trial registries) for included studies will be reviewed to determine whether outcomes of interest were assessed but not reported in the identified publication. For older studies where trial registration was not mandatory, authors will be contacted to determine whether data on the outcomes of interest to the review are available for provision to the systematic review team. The abstracts of included studies not published in full using English will be reviewed for potential for data extraction.

Based on the results of the eligibility step, included studies will be divided into categories, according to the primary health behavior or lifestyle target of the intervention (tobacco, physical activity, diet quality, sleep-wake disturbance, and alcohol/other drugs). Included studies on weight loss interventions will be reviewed for information about the primary target of behavior within the weight loss intervention (e.g., physical activity, diet). We expect that, within each lifestyle intervention/behavior, a range of outcomes will be reported (e.g., minutes or hours of moderate or vigorous activity per day or week). We will extract data related to all reported outcomes for each included study for this review. Studies reporting more than one behavioral outcome will contribute data to all relevant intervention analyses.

Data will be extracted from all eligible studies using a data extraction form specifically developed for this study (see Additional file 1). Extracted data will include study design, study setting, sample size, study population and participant demographics (including diagnoses), baseline characteristics (including phase of bipolar disorder), recruitment and retention rates (including completion), details of the intervention, details of health behavior outcomes (all reported timepoints), details of other reported study outcomes (e.g., quality of life, BD symptoms), indicators of acceptability to participants, and information for the assessment of the risk of bias. All reported trial outcomes related to the lifestyle behavior targeted by the intervention will be extracted in the event that a study does not report on the primary outcome for the review but reports on a structurally/conceptually similar outcome related to the primary outcome (possible reporting bias).

Seven review authors (LT, JL, FJ, TH, AT, SH, and FK-L) will independently extract data from eligible studies using the standardized form, with discrepancies identified and resolved through discussion with an eighth team member (LS). Missing data will be requested from study authors, who will be asked to supply the missing information in de-identified, summarized format. Included studies will be divided between the five reviewers, such that each study has data extracted by three reviewers.

The Cochrane Collaboration’s tool for assessing risk of bias will be used to conduct a risk of bias (quality) assessment for individual studies included in the review [25]. As per this tool, criteria for judging risk of bias will be applied (low, unclear, high) to the following domains: selection bias (random sequence generation, allocation concealment); performance bias (blindness of participants and personnel); detection bias (blindness and modality of outcome assessment); attrition bias (incomplete outcome reporting); and reporting bias (selective outcome reporting). A similar approach will be taken to non-randomized studies included in the review, using the eight-item Newcastle-Ottowa Scale as a guide [26]. To determine reporting bias, the ORBIT classification system will be adopted [27] for each included study. In each intervention category (tobacco, physical activity, diet quality, sleep-wake disturbance, alcohol/other drugs), a matrix will be constructed for included studies, which evaluates the reporting of the primary outcome for the review, and other trial outcomes related to that lifestyle behavior. Each reported outcome will be given a rating according to whether the primary outcome/other outcome was reported in full (with statistical analysis results reported in full), not reported at all, or partially reported (e.g., only the *p* value was provided). For those studies given a “partially reported” or “not reported” rating for the given primary outcomes of interest, the nine-point ORBIT classification system will be applied. Each of these studies will be given a classification of A-I, depending on the clarity with which the primary outcome was measured and analyzed. Clarification will be sought from trial registry information and/or study authors to determine whether a study might be classified as incomplete outcome reporting and at risk of outcome reporting bias (due to a change in outcomes from registered details).

### Primary outcomes

The primary outcomes for the review will be changes in the lifestyle behaviors targeted by the intervention between baseline and the immediate post-intervention follow-up assessment, namely tobacco use, levels of physical activity, diet quality, sleep quality, alcohol use, and illicit drug use. In order to synthesize results across all included studies within a particular lifestyle behavior, a review of reported outcomes for each included study will be conducted by LT and FK-L to determine how the primary outcome of interest is reported for each study in each lifestyle area (e.g., abstinence rates for tobacco use studies versus cigarettes per day, minutes of vigorous exercise per week for physical activity studies versus hours per week of non-leisure sedentary behavior). Collaborating researchers specializing in each of the lifestyle categories will be consulted during this process to ensure that the primary outcome for each category best represents the available data and is relevant and important to the field (tobacco = AB, FK-L, SW; physical activity = RC, SD, SW; diet quality = FJ, AT, SW; sleep-wake disturbance = NR, SH, TH, SW; alcohol/other drugs = AB, FK-L, SW). SW, a consumer-researcher, will review these primary outcomes for relevance and importance to people experiencing BD.

### Data analysis

The primary analysis (estimated intervention effect) for the review will be based on all studies, regardless of risk of bias, but presented with a description of the risk of bias in individual domains.

We will provide a narrative synthesis of the findings from the included studies, based on the framework reported by Popay et al. [28]. Separate narratives will be presented for each lifestyle domain: tobacco, alcohol, illicit drugs, sleep, physical activity, and healthy eating. We will describe the theoretical basis of the interventions for included studies, which includes discussion about what works, for whom, and why. We will provide summaries of intervention effects in a tabular form, using data from the extraction phase, organized according to primary outcome for that lifestyle domain, and other reported outcomes at all reported timepoints. We will also report on studies in each lifestyle area according to the type of intervention (single, multi-focus, eHealth), target population characteristics (including phase of bipolar disorder), type of outcome (including objective, self-report, clinician-rated), intervention content, and the phase of BD in which the intervention was delivered (manic/hypomanic, depressed, euthymic). We will calculate and report risk ratios (for dichotomous outcomes such as abstinence from tobacco) or standardized mean differences (for continuous outcomes such as minutes of physical activity). Forest plots will be generated for the primary outcome in each lifestyle area.

Where there are two or more randomized controlled trials in a particular lifestyle area, we will carry out a meta-analysis of primary outcome using these studies. We anticipate that there will be limited scope for meta-analysis across all lifestyle domains. The exception may be for smoking cessation where there is likely to be a larger pool of completed studies in BD. The meta-analysis will build on the narrative synthesis in the eligible lifestyle area by combining primary outcomes across studies to provide an overall summary estimate of effect of the lifestyle intervention. Both fixed and random effects models will be applied, stratified by study size, and overall summary estimates of effect added to the forest plots for that lifestyle area.

In interpreting the results of the analysis, the Grading of Recommendations Assessment, Development, and Evaluation system (GRADE) will be applied to determine the quality of evidence for each intervention target reported in the review [29]. The GRADEpro [30] program will be used to facilitate this analysis. We will use the following in grading the quality of the evidence arising from the systematic review for each of the intervention targets in bipolar disorder:

- High = further research is very unlikely to change our confidence in the estimate of effect.
- Moderate = further research is likely to have an important impact on our confidence in the estimate of effect and may change the estimate.
- Low = further research is very likely to have an important impact on our confidence in the estimate of effect and is likely to change the estimate.
- Very low = any estimate of effect is very uncertain.

## Discussion

In the general population, eliminating health risk behaviors has the potential to prevent 80 % of heart disease, stroke, 80 % of type 2 diabetes, and 40 % of cancers [31]. The same is likely to be true in mental illness. Given the complex nature of bipolar disorder, uncertainty exists about the most effective way to drive improvements in health behaviors, as treatments effective in other populations may not directly and safely translate to this patient population [13]. The planned review will synthesize and evaluate the available evidence regarding the behavioral or psychosocial treatment of lifestyle-related behaviors in people with BD. From this review, we will identify gaps in our existing knowledge and research evidence about the management of unhealthy lifestyle behaviors in people with BD. We will also identify potential opportunities to address lifestyle behaviors in BD, with a view to reducing the burden of physical ill-health in this population.

## Abbreviation

CVD, cardiovascular disease

## Additional file

Additional file 1: Systematic review data extraction form. (PDF 75 kb)
