# Supplementary material for: Study protocol for a systematic review of evidence for lifestyle interventions targeting smoking, sleep, alcohol/other drug use, physical activity, and healthy diet in people with bipolar disorder
Source: Syst Rev. 2016 Jul 5;5:106. doi: 10.1186/s13643-016-0282-9 (PMC4932766; doi:10.1186/s13643-016-0282-9)
Supplement: Additional file 1: — Systematic review data extraction form. (PDF 75 kb) [file 13643_2016_282_MOESM1_ESM.pdf]

Systematic Review
Data Extraction Form

Study Details

Study ID: Reviewer initials:

First author surname, initial: Year of publication:

Journal name:

Methods (20)

Study design: Study setting:

Sample Size: Study population:

Intervention target:
(tobacco, physical activity, diet quality, sleep-wake disturbance, alcohol, cannabis, illicit drug)

Type of intervention:

SELECTION BIAS (low, unclear, high):

Random sequence generation:

Allocation concealment:

PERFORMANCE BIAS (low, unclear, high):

Blinding of participants and personnel:

DETECTION BIAS (low, unclear, high):

Blinding of outcome assessment:

Mode of outcome assessment:

ATTRITION BIAS (low, unclear, high):

Approached = Eligible = Consented = Baseline =

Intervention completion rates =

Follow-up 1= Follow-up 2 = Follow-up 3 =

Incomplete outcome data short term?

Incomplete outcome data long term?

Results (20)

REPORTING BIAS (low, unclear, high):

Primary outcome, incl. endpoint:

Other outcomes, incl. endpoint:

Phase of bipolar disorder for participants at recruitment:

Age at baseline: Gender split:

PRIMARY OUTCOME:

Data analysis approach: Management of missing data:

Statistical results – Coefficient: 95% CI: p-value:

Adjusted for:

OTHER OUTCOMES: (detail)

Statistical results – Coefficient: 95% CI: p-value:

Adjusted for:

OTHER OUTCOMES: (detail, and add as necessary)

Statistical results – Coefficient: 95% CI: p-value:

Adjusted for:

Multiple health behavior outcomes reported? (Y/N)

Details of multiple health behavior outcomes:

Medication side effects reported: (detail)

Patient acceptability reported: (detail)

Main/Additional Findings:

Study limitations:

Selective outcome reporting? (rating A-I):
(as per ORBIT classification system, 21)
